# Evaluation of a home treatment approach to schizophrenia in rural Pakistan: the SOUL Programme

**DOI:** 10.1192/j.eurpsy.2022.1570

**Published:** 2022-09-01

**Authors:** S. Raja, F. Soomro, B. Junejo, R. Wagan, S.S. Afghan

**Affiliations:** 1 John Radcliffe Hospital, Neurology, Oxford, United Kingdom; 2 Shaheed Mohtarma Benazir Bhutto Medical University, Psychiatry, Larkana, Pakistan; 3 Dorothy Pattison Hospital, Adult Mental Health, Walsall, United Kingdom

**Keywords:** schizophrénia, Outreach, Mixed Methods, LEDC

## Abstract

**Introduction:**

Psychiatric services in LEDCs face a tripartite challenge: (i) limited financial capital; (ii) scarcity of professionals; (iii) restrictive health beliefs. Inevitably, services developed for the first-world are ill-suited here. Psychiatric services must be designed from the ground up: inspired by but not a replica of best practices in the developed world. The SOUL project in Larkana, Pakistan provides home based assessment by a psychiatrist and fortnightly treatment by a mobile nursing team for schizophrenic patients. Psychoeducation of carers and the community as well as facilitation of work for patients are core aims. This mixed-methods study evaluates the experiences of primary stakeholders - patients and their carers.

**Objectives:**

1.Are patients and carers satisfied with the care received? 2.Has SOUL been successful in changing health beliefs? 3.How could the programme be improved?

**Methods:**

The principal investigator accompanied the team for 4-weeks. Purposive sampling was employed. Satisfaction was assessed quantitatively using the likert based PSQ-18 questionnaire. Thereafter, qualitative data was gathered using semi-structured interviews and analysed using a grounded theory approach. A total of 27 interviews were conducted before data saturation.

**Results:**

100% of interviewees answered ’Satisfied’ or ’Very’ Satisfied to all elements of the PSQ-18. Above all, stakeholders valued that treatment was free and highly accessible (home visits), promoting treatment adherence. They felt psychoeducation events significantly reduced community stigma and made families more likely to seek psychiatrists over faith healers. Provision of respite care was suggested as a future improvement.

**Conclusions:**

SOUL is highly valued by stakeholders and offers an excellent example of LEDC psychiatric care.

**Disclosure:**

No significant relationships.

